# Diagnosis and treatment of left ureteral injury as a rare complication of oblique lumbar interbody fusion surgery: a case report and literature review

**DOI:** 10.1186/s12894-022-01070-z

**Published:** 2022-09-08

**Authors:** Weiheng Wang, Bing Xiao, Xiaodong Huang, Jiangming Yu, Yanhai Xi, Guohua Xu, Xiaojian Ye

**Affiliations:** 1 Department of Orthopaedics, Second Affiliated Hospital of Naval Medical University, Shanghai, 200003 China; 2 Department of Orthopedics, The Third Affiliated Hospital of Guangzhou Medical University, Guangdong, 510140 China; 3 Department of Orthopedics, Tongren Hospital, Shanghai Jiao Tong University School of Medicine, Shanghai, 200336 China

**Keywords:** Oblique lumbar interbody fusion (OLIF), Ureteral injury, Complications, Case report

## Abstract

**Background:**

Oblique lumbar interbody fusion (OLIF) surgery has been performed as a minimally invasive lateral lumbar fusion technique in recent years. Reports of operative complications of OLIF are limited, and there are fewer reports of ureteral injuries.

**Case presentation:**

A 62-year-old Chinese woman diagnosed with "lumbar spondylolisthesis (L4 forward slip, I degree)" underwent OLIF treatment. The surgical decompression process was smooth, and the cage was successfully placed. After the expansion sleeve of OLIF was removed, clear liquid continuous outflow from the peritoneum was found. The patient was diagnosed with a ureteral injury. The urological surgeon expanded the original incision, and left ureteral injury anastomosis and ureteral stent implantation were performed. The patient was changed to the prone position and a percutaneous pedicle screw was placed in the corresponding vertebral body. The patient was indwelled with a catheter for 2 weeks, and regular oral administration of levofloxacin to prevent urinary tract infection. After 2 months, the double J tube was removed using a cystoscope. One year after surgery, the symptoms of lumbar back were significantly improved, and there were no urinary system symptoms. However, the patient needed an annual left ureter and kidney B-ultrasound.

**Conclusion:**

Ureteral injury is a rare complication and is easily missed in OLIF surgery. If the diagnosis is missed, the consequences can be serious. Patients should undergo catheterization before the operation and hematuria should be observed during the operation. We emphasize the careful use of surgical instruments to prevent intraoperative complications. In addition, after withdrawing the leaf in the operation, it is necessary to carefully observe whether a clear liquid continues to leak. If ureteral injury is found, one-stage ureteral injury repair operation should be performed to prevent ureteral stricture.

## Background

Oblique lateral lumbar interbody fusion (OLIF) is a minimally invasive lumbar interbody fusion technique through an expandable channel that can be used to treat lumbar spondylolisthesis and lumbar kyphosis. The procedure has the advantages of simple operation, minimal trauma and quick recovery [1, 2]. OLIF uses the physiological space between the retroperitoneal abdominal vascular sheath and the anterior border of the psoas muscle as the access channel, which can avoid the excessive traction of the frontal large vessels and other tissues by anterior lumbar interbody fusion surgery (ALIF). Furthermore, it can avoid the destruction of the lumbar plexus and psoas muscle by lateral lumbar interbody fusion (LLIF). In theory, the complications associated with the approach can be minimal [3–5]. In Japan, 1150 patients with OLIF surgery had a total complication rate of 15.3%. The incidence of ureteral injury was 0.3%, which was found after abdominal discomfort, and the proportion of OLIF ureteral injury was higher than that of ALIF/LLIF [6]. There are few case reports on OLIF surgery with ureteral injury, and ureteral injury has been found after surgery [7, 8]. This article reports a case of ureteral injury found during OLIF surgery, and reviews the literature on how to prevent, diagnose and treat this complication.

## Case presentation

**Fig. 1:**
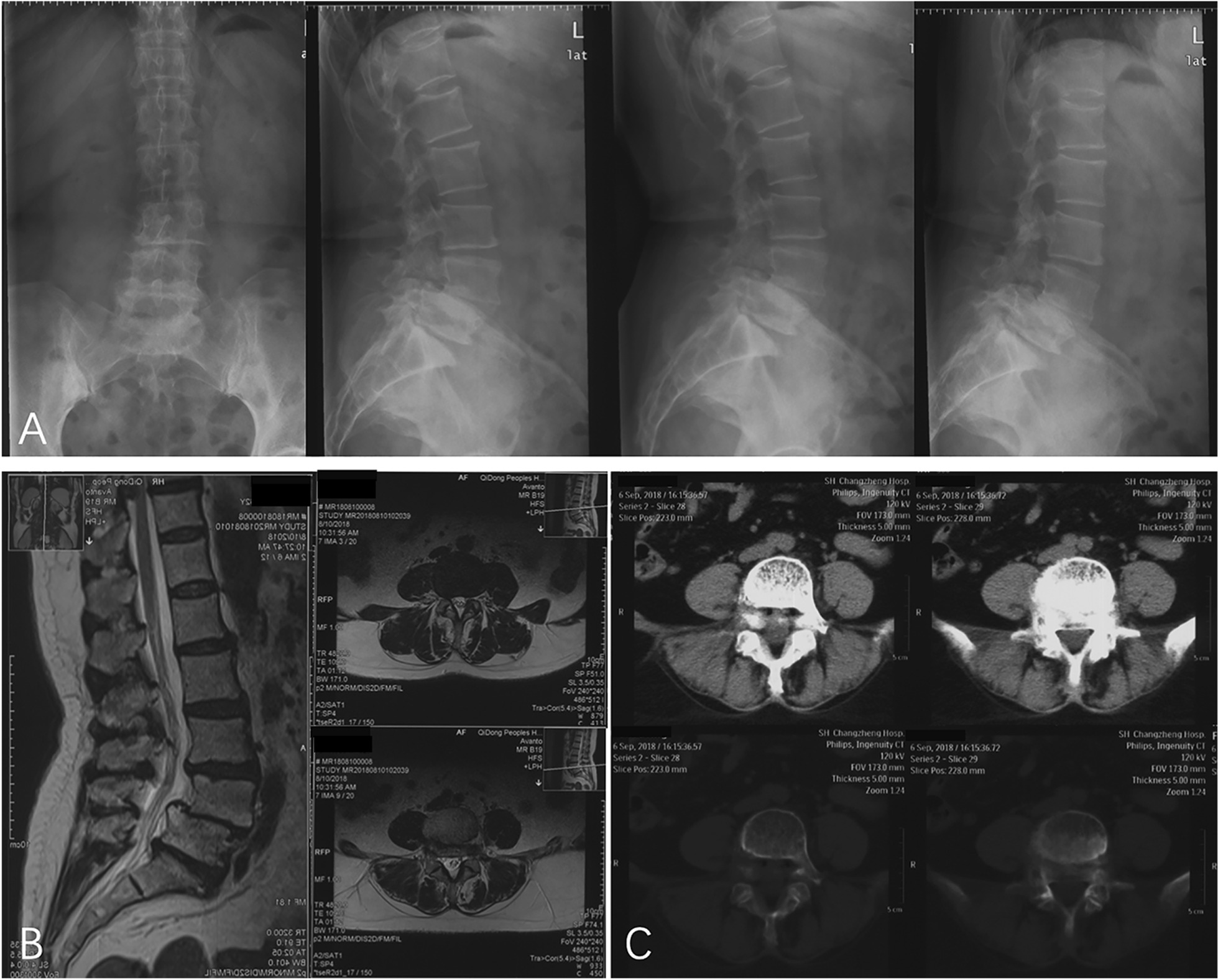
Preoperative radiography lumbar vertebrae, lateral position, overextension (**a**), MRI (**b**) and CT (**c**) revealed L4 vertebral body slip forward (I degree) and L4/5 lumbar spondylolisthesis

A previously healthy 62-year-old woman (BMI = 28.1) experienced progressive low back pain with bilateral hip pain soreness for the past 1 year. The patient's symptoms did not improve significantly after conservative treatment. She complained of back and pelvic pain, which had persisted for the past year. The pain in the lower back was aggravated when standing for a long time and when tired, and the pain was relieved when resting. Japanese orthopaedic association (JOA) score was 19 points, and the low back pain visual analogue scale (VAS) score was 5 points. The neurological examination was normal. Lumbar X-ray positive lateral position, lateral and overextension, overcurved slices showed lumbar 4 (L4) vertebral body instability and slipped forward I degree (Fig. 1a). Magnetic resonance imaging (MRI) revealed lumbar degeneration and L4 vertebral body slip forward (I degree), corresponding to segmental spinal stenosis, spinal cord and nerve root compression (Fig. 1b). Lumbar computed tomography (CT) showed: lumbar vertebrae 4 degenerative spondylolisthesis, no fractures in the lumbar isthmus (Fig. 1c). According to the patient's symptoms, signs and imaging findings, the patient was diagnosed with lumbar degenerative spondylolisthesis (I degree). After completing the preoperative routine examination to eliminate contraindications, the patient underwent elective OLIF surgery under general anesthesia.

**Fig. 2:**
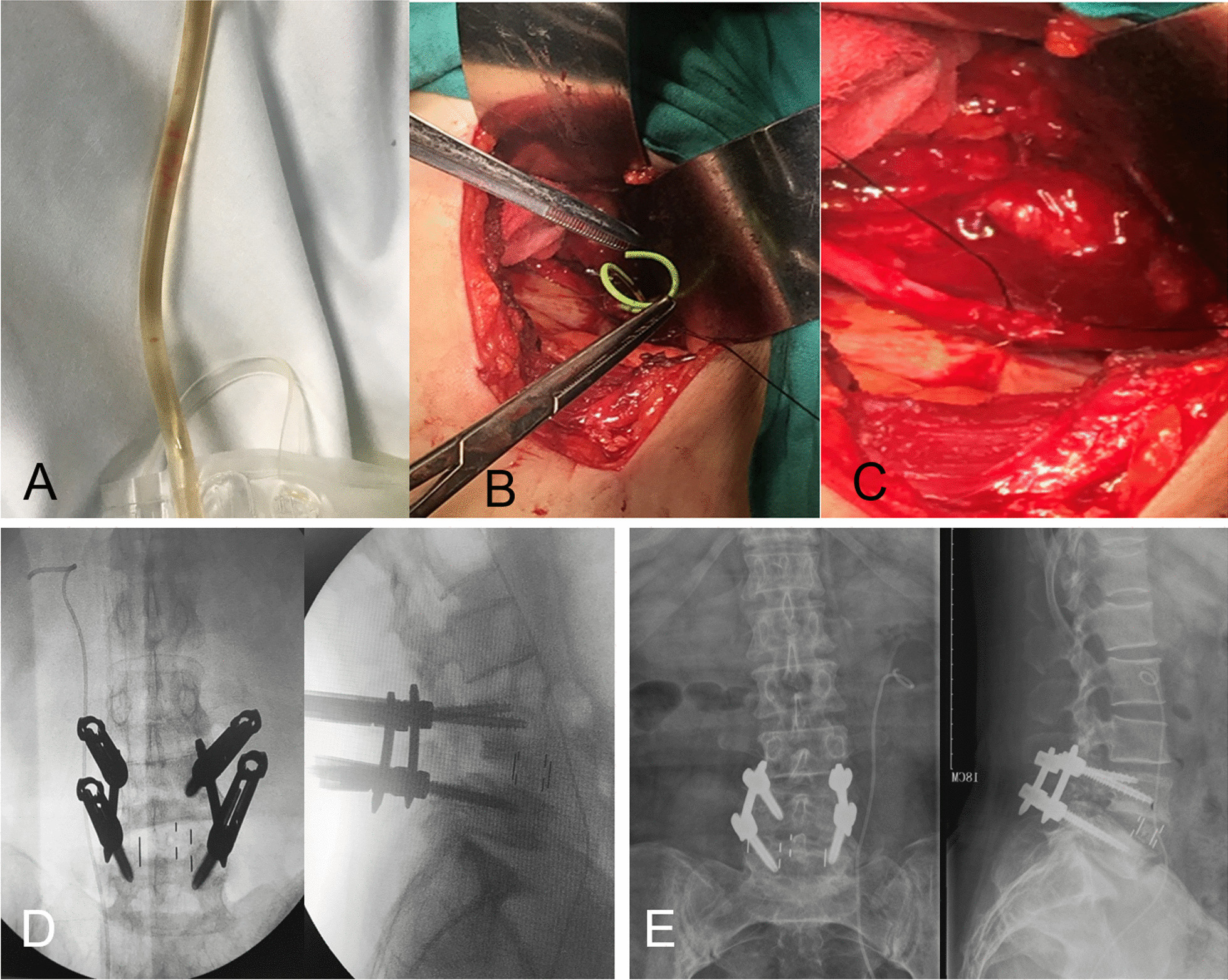
Photos of left ureter repair during surgery and postoperative review imaging. **a** Showed that hematuria was found in the catheter. **b** Showed that the ureteral stent was inserted into the left ureter during the operation. **c** Showed that the left ureter was repaired during surgery. **d** Showed that the intraoperative X-ray indicated the height recovery of the intervertebral disc and the left ureter double J tube was in good position. **e** Showed that a good fixation position within 1 year after surgery

Under general anesthesia, the patient underwent routine catheterization, and was placed in the right lateral position. Then the skin was shaved and prepared with povidone iodine and draped in a sterile manner. With the L4/L5 intervertebral space as the center, a vertical incision (approximately 4 cm) was made at the level of the left anterior axillary line. The skin and subcutaneous tissue were cut, and the muscle tissue was bluntly separated layer by layer. The peritoneal fat was pushed forward to protect the viscera and other tissues until it touched the psoas muscle. The psoas muscle was pushed backward and the guide needle was placed on the leading edge of the psoas muscle. The expansion cannula was placed step by step, and a fixation screw was placed in the L4/L5 intervertebral space. When placing the self-retaining retractor onto the L4/L5 intervertebral disc, a thread pin, which fixates the retractor through a small hole, went deep into the anterior space of the spinal column. After confirming that the CAGE position was good during the operation, the expansion sleeve was removed, and a large amount of saline was used to rinse the incision. Then double-click electrocoagulation was used to stop the bleeding. It was found that there was a clear liquid flowing out of the peritoneum at the level of the L4/5 segment, and hematuria was found in the catheter (Fig. 2a). These phenomena indicated that the left ureter may have been damaged. A urinary surgeon was consulted urgently during the operation, and left ureteral injury was definitively diagnosed. Then the patient underwent left ureteral injury exploration and ureteral stent (double pigtail stent, Cook, USI-626-B) placement into the ureter (Fig. 2b, c). The original incision was expanded along about 6 cm, and the shape of the left ureter was explored. There was a ureteral leak at the level of the L4/L5 segment. Both ends of the ureteral injury were freed, and the F4.5 short-term ureteral stent (double pigtail stent, cook, USI-626-B) was implanted from the ureteral leak. A 5–0 absorbable thread was used to suture the ureteral end without tension of the ureter. Then, the abdominal incision was closed, and the patient was changed to the prone position. Under the guidance of the C-arm, percutaneous pedicle screws were placed into the L4 and L5 bilateral pedicles. The intraoperative plain film showed the height recovery of the intervertebral disc and the left ureter stent was in a good position (Fig. 2d). The left ureteral stent was removed 2 months after surgery, and the lumbar spine X-ray showed good spine stability 1 year after surgery (Fig. 2e).

The patient was indwelled with a catheter for 2 weeks, and regular oral administration of levofloxacin to prevent urinary tract infection. After 2 months, the ureteral stent was removed by using cystoscope. One year after surgery, the symptoms of lumbar back were significantly improved (JOA score of 25 point, low back pain VAS score of 1 point), and there were no urinary system symptoms 3 years after surgery. However, the patient still needs to regularly check the left ureter and the left kidney by ultrasound annually.

## Discussion and conclusions

Ureteral injury is a rare and serious complication of OLIF surgery and is rarely reported, requiring rapid diagnosis and intervention to avoid kidney failure [6–8]. Shunsuke et al. [6] reported an incidence of 0.3%. Anand et al. [7] reported three cases of urological injury, including two ureteral injuries and one kidney injury. Ureteral injury had also been reported in anterior [9], posterior [10, 11] and lateral [8] lumbar surgeries. The OLIF operation needs to pass the peritoneum, push the psoas muscle back, and use an expansion sleeve to open the channel, therefore, the probability of damage to the ureter is higher than that of LLIF. A large number of cases studies in Japan showed that the incidence of ureteral injury after OLIF surgery (0.3%) was higher than that after LLIF (0.1%) [6]. Because ureteral injury has no obvious symptoms in OLIF, it is easy to miss the diagnosis during the operation. All current reported cases of OLIF were diagnosed by postoperative imaging due to abdominal discomfort and hematuria. The reason for missed diagnosis during the operation is that the OLIF incision is small and deep, and the field of view is limited. In addition, the spine surgeon is relatively new to the structure of the retroperitoneum. Missed diagnosis of ureteral injury may lead to serious complications such as ureteral stricture, hydronephrosis, and even renal failure.

**Fig. 3:**
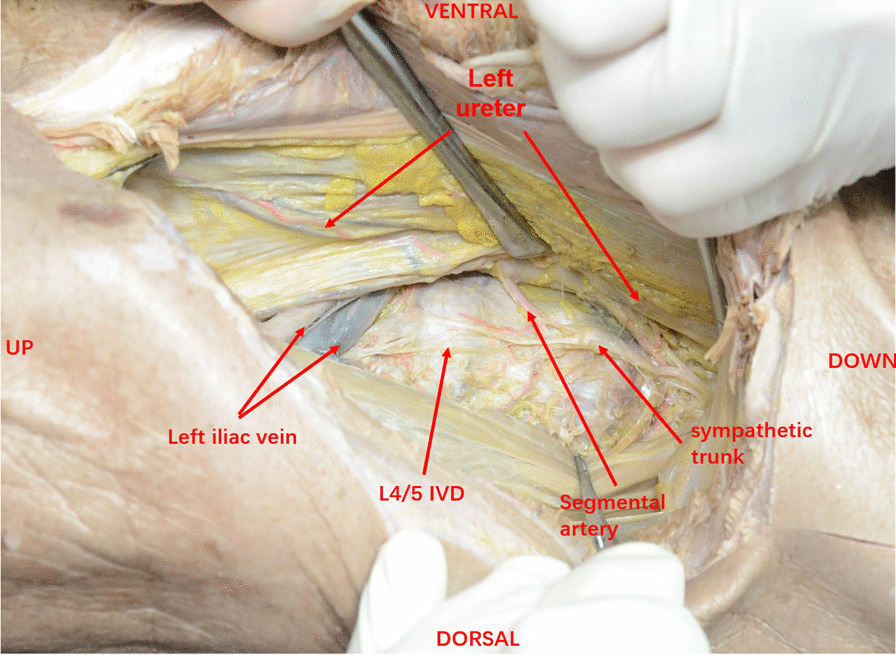
The adjacent relationship of the structures under the OLIF approach

Ureteral injury mainly occurs in pelvic and abdominal surgery. According to the literature, the incidence is less than 4% [12],of which hysterectomy is the majority (54%), followed by colorectal surgery (14%), and pelvic surgery such as ovarian tumor resection (8%) and abdominal vascular surgery (6%); in contrast, orthopedic surgery combined with ureteral injury is rare [13]. Anatomically, the lower lumbar ureter lays immediately anterolateral to the L4–L5 interspace, directly on the anterior longitudinal ligament between the vertebral body and the lumbar muscle. When the psoas muscle is pushed back, the ureter may be damaged, especially when the lumbar muscle hypertrophy is difficult to push back (Fig. 3). The anatomic relationship between the ureter and OLIF access studied by computed tomographic urography showed that [14]: the bilateral ureters progressively descents from the lateral margin of the psoas major muscle to the anteromedial margin. The range of bilateral surgical access for OLIF progressively decreases from L2/L3 to L4/L5, and the left-sided surgical path is larger than the right-sided surgical path at the same level. Ureters at the right-sided L3/L4 level and bilateral L4/L5 levels are at high risk for operative injury. In particular, the risk of right-sided ureteral injury in L4/L5 is the highest. However, the effect of the right lateral position and general anesthesia on the anatomic relationship between the ureter and OLIF access remains to be further studied.

Early diagnosis is critical for the prognosis of complications associated with ureteral iatrogenic injury [15], with earlier diagnosis, leading to a simpler procedure for remediation. The ideal time for ureteral repair is when damage occurs. More than 3 months after ureteral injury, ureteral end-to-end anastomosis and ureteral bladder anastomosis are usually required [16]. Ureteral rupture injury often manifests as a continuous clear liquid in the surgical field, and sometimes a tubular broken end or a rupture can be seen. Sometimes gross hematuria can be seen in urethral catheters. Therefore, during OLIF surgery, the surgeon should pay attention to whether the peritoneum has clear liquid continuous exudation, especially after removing the expansion sleeve. Another sign of ureteral damage is gross hematuria. Therefore, patients with OLIF should be routinely catheterized before surgery, and the color of urine in the catheter should be closely observed during the operation. Once ureteral injury is suspected during the operation, a urological surgeon must be asked to perform an emergency consultation during the operation to assist the diagnosis and treatment.

One of the more reliable methods for any operation that suspects ureteral injury is to inject 5 ml of indigo carmine or methylene blue into the catheter. Then observe whether there is fuel leakage around the ureter, if there is, the ureteral injury at the place is definitely diagnosed [17]. However, the missed diagnosis of ureteral injury in OLIF surgery is relatively common [18]. Most ureteral injuries have no obvious abnormality due to intraoperative expansion channel compression, and they are missed because of the lack of experience of the surgeon. Patients with fever, ipsilateral low back pain, persistent flatulence, ascites, hematuria or anuria after OLIF should be highly suspected of ureteral injury. Postoperative evaluation of ureteral injury diagnostic tests included laboratory tests (urine routine, serum creatinine, renal function, etc.), cystoscopy, and imaging techniques. It has been reported that serum creatinine increases by 71–88 µmol/L 24–72 h after unilateral ureteral ligation [19]. Intravenous pyelography (IVP) is useful for assessing hydronephrosis, unilateral renal function, and continuous integrity of the ureter. However, 7% of cases after ureteral injury show normal results by IVP [20]. Retrograde ureterography is almost 100% accurate in diagnosing ureteral injury. It can show ureteral injury obstruction and clear fistula formation. Abdominal CT, urinalysis, and cystoscopy are essential in diagnosis, and can assist retrograde angiography in confirming the diagnosis. B-ultrasound has no advantage in showing ureteral injury, but can suggest the presence or absence of hydronephrosis. The late clinical manifestations of ureteral injury are urinary cysts, ureteral fistula formation (such as vaginal, intestinal, or skin leakage), secondary to hydronephrosis, and renal atrophy [20].

Pathophysiological studies have found that the new transitional epithelium of the ureteral anastomosis begins to appear after 2 weeks, and ureteral function recovers after 4 weeks [17]. After ureteral repair, if the drainage around the ureter is less than 30 ml per day, the drainage tube around the ureter can be removed. If the bladder is opened during the repair operation, the catheter needs to be placed for 7–10 days. After the urethral catheter is removed, the urethral catheter needs to be replaced when the drainage around the ureter increases. If the drainage around the ureter does not increase significantly, the drainage tube around the ureter can be removed. The double J tube for internal ureteral drainage generally needs to be placed for 3–6 weeks to ensure that the function of the ureter is repaired. After removal of the ureteral stent, the ureter and kidney B ultrasound should be followed up every 3–6 months [20, 21]. During follow-up, if hydronephrosis, ureteral hydrops, or even renal function damage occurs, the urologist must further confirm whether there is ureteral stenosis. If this happens, the patient needs standardized treatment for ureteral stenosis. Early diagnosis and primary repair operations are key factors to prevent ureteral stenosis after surgery.

The best treatment for ureteral injury is prevention. The surgeon should be familiar with the anatomical relationship of the ureter in the posterior peritoneum. Before surgery, the patient’s preoperative imaging data should be carefully read, and the relationship between the urinary tube and the psoas major should be carefully distinguished. For patients with high risk factors for ureteral injury before surgery, contrast-enhanced computed tomographic urography can be routinely performed to clarify the location of the ureter. Patients should undergo routine catheterization before OLIF, and OLIF should have good exposure during surgery. If the exposure is not satisfactory, the incision should be extended appropriately. The operation should be meticulous, and avoiding a blind operation is the key to preventing ureteral injury. If major bleeding occurs, the wound should first be pressurized to stop the bleeding. The use of electrocautery or electrocoagulation to operate on the retroperitoneal structure should be avoided. After recognizing the tissue clearly, the bleeding blood vessel should be sutured to avoid tying the ureter in large tissues. After the expansion channel is removed, it is necessary to carefully observe whether the retroperitoneal structure has a clear liquid that continues to flow out to prevent missed diagnosis. Before closing the incision, one should carefully observe whether there is gross hematuria in the catheter. Relative contraindications for OLIF surgery are those with a history of urinary tract or suspected urinary abnormalities, and patients with a history of retroperitoneal tumors, infections and surgery. If these patients select OLIF, intravenous urography or contrast-enhanced computed tomographic urography should be performed before surgery. If necessary, a ureteral stent should be placed before surgery to reduce the risk of missing a ureteral injury.

Ureteral injury is a rare complication of OLIF, which is characterized by a low incidence, a high rate of missed diagnosis, and great harm after missed diagnosis. The indications and contraindications of OLIF should be considered strictly. The surgeon should be familiar with the anatomical relationship of the ureter in the posterior peritoneum, and carefully read the patient's preoperative imaging data before surgery. Blind operation should be avoided. An electrosurgical knife, electrocoagulation and ligation should be used with caution during OLIF. The prevention of ureteral injury is key. Careful observation during the operation should be performed to prevent missed diagnoses. If ureteral injury is suspected during the operation, urologist should be promptly consulted for a timely diagnosis. If the diagnosis is confirmed during the operation, one-stage surgical repair should be performed. The diagnosis can be confirmed after the operation, one-stage repair surgery should be performed as much as possible, and the second-stage repair should be performed for those that cannot be repaired in the first stage. Patients need regular follow-up after surgery to examine renal function and urinary B-ultrasound.

## Data Availability

The datasets used in this study are available from the corresponding author on reasonable request.

## Abbreviations

OLIF: Oblique lumbar interbody fusion
ALIF: Anterior lumbar interbody fusion surgery
LLIF: Lateral lumbar interbody fusion
JOA: Japanese orthopaedic association
VAS: Visual analogue scale
MRI: Magnetic resonance imaging
CT: Computed tomography
IVP: Intravenous pyelography
